# Who pays for home care? A study of nationally representative data on disabled older Americans

**DOI:** 10.1186/s12913-015-0978-x

**Published:** 2015-07-31

**Authors:** Alexander L. Janus, John Ermisch

**Affiliations:** School of Social and Political Science, University of Edinburgh, Chrystal Macmillan Building, 15a George Square, Edinburgh, EH8 9LD UK; Department of Sociology, University of Oxford, Manor Road Building, Manor Road, Oxford, OX1 3UQ UK

**Keywords:** Home and community based care and services, Disabilities, Healthcare policy, Intergenerational relationships

## Abstract

**Background:**

We examine who pays for services that support disabled older Americans at home. We consider both personal sources (e.g., out-of-pocket payment, family members) and publicly funded programs (e.g., Medicaid) as sources of payment for services. We examine how the funding mix for home care services is related to older people’s economic resources, needs for care, and other socio-demographic characteristics.

**Methods:**

Our sample consists of 11,725 person-years from the 1989, 1994, 1999, and 2004 waves of the National Long-Term Care Survey. Two-part regression analyses were performed to model hours of care received from each payer. “Random effects” and “fixed effects” estimation yielded similar results.

**Results:**

About six in ten caregivers (63 %) providing home care services are paid by personal sources alone. By contrast, 28 % receive payment from publicly funded programs alone, and 9 % from a combination of personal and public program sources. Older people with family incomes over 75,000 dollars per year receive 8.5 more hours of home care overall than those in the lowest income category (less than 15,000 dollars). While the funding mix for home care services is strongly related to older people’s economic resources, in all income groups at least 65 % of services are provided by caregivers paid in whole or in part from personal sources. In fact, almost all (97 %) home care received by those with family incomes over 75,000 dollars per year are financed by personal sources alone.

**Conclusions:**

We outline the implications that heavy reliance on personally financed services and economic disparities in overall services use has for disabled older Americans and their families.

**Electronic supplementary material:**

The online version of this article (doi:10.1186/s12913-015-0978-x) contains supplementary material, which is available to authorized users.

## Background

Home care services (provided by paid caregivers) are a valuable source of post-acute and long-term care for older people and their families. These services may help older people with health problems remain in the community [1], while complementing or serving as a substitute for family caregiving [2]. For these reasons, ensuring equitable access to home care services has been an important goal of long-term care policy, both in the United States and abroad. Understanding the ability of those who are not adequately covered by publicly funded programs to access alternative sources of payment for services is essential to evaluating the success of policy in promoting equitable access. Previous research based on US data provides an informative profile of users of publicly funded home and community-based services (HCBS) and reveals socio-demographic characteristics that are associated with the take-up of these services [3, 4]. However, with few exceptions, studies have not distinguished between home care services by who pays for them and have not examined how publicly funded home care services interface, or combine, with services paid for by various personal sources (see Liu et al. [5] and Spector et al. [6] for exceptions).

Considering the situation in the United States, we ask three questions. First, which socio-demographic groups are least likely to receive home care services paid for by publicly funded programs—namely, Medicare and Medicaid? Second, to what extent are older people who are not covered by relevant public programs able to access services financed by other means (e.g., out-of-pocket payment)? Third, how is older people’s use of home care services from each source related to indicators of their needs for care and the availability of informal family care? To address these questions, we use detailed data on home care services use, different payment sources, and socio-demographic characteristics of potential recipients from the 1989, 1994, 1999, and 2004 waves of the National Long-Term Care Survey (NLTCS).

While family caregivers are responsible for the majority of home care that older people receive across countries, home care services provided by paid caregivers nonetheless represent a significant source of help. Estimates for the United States suggest that 18 % of older people aged 65 and older with long-term care needs receive at least some help from paid caregivers. Among those not living with family members, about one-quarter (26 %) receive help from paid caregivers [7].

Medicare and Medicaid are the most important payers of long-term care in the United States. In 2013, of the total $235.6 billion in estimated spending for nursing home and home health care in the United States, Medicare paid for 29 % and Medicaid paid for 32 % [8]. Each program pays for at least part of the services received by about one-third of the 1.6 million community residents receiving paid long-term care [7]. Despite some broad similarities, there are important differences between these two programs with regard to how each program is financed, the populations that are eligible for and receive services, and the types of services that each program covers.

Both Medicare and Medicaid were created in 1965 as amendments to the Social Security Act. Medicare is a federal program, while Medicaid is a state-managed program that is jointly financed by the federal government and states. Both programs offer home and community-based services (HCBS) to eligible older people. Medicare provides post-acute home health care (after hospitalization) to older people in need of skilled care or therapy services for short periods [9, 10]. By contrast, while the only mandated HCBS under Medicaid are home health care for people who are eligible for institutional care, most states offer additional services through two optional programs: 1915(c) HCBS waivers and the state plan personal care services benefit [11]. Personal assistance services such as hands-on assistance with personal care and household management tasks are primarily covered by these optional programs [12].

Similar to some other state-funded programs that provide HCBS, Medicaid targets the economically disadvantaged [13, 14]. Medicaid targets individuals based on both income and asset tests. Eligibility limits vary by state. Some states apply the federal thresholds for receipt of Supplemental Security Income, which in 2005 (the most recent year in our analysis) were 869 dollars per month in countable income and 3000 dollars per month in countable assets for couples. In other states Medicaid pays for long-term care services for those with incomes up to 300 % of the federal Supplementary Security Income threshold [13]. State-funded programs generally seek to reach a broader population of older people than that targeted by Medicaid, but Medicaid spending in most states dwarfs spending on state programs [14]. Functional need eligibility criteria and cost control measures further restrict the scope of Medicaid HCBS, and similar to the financial eligibility limits, vary considerably by state [11]. Due in part to these eligibility limits, the adult population served by Medicaid is considerably more likely to have incomes below the poverty level and to report fair or poor health status than privately insured adults [15].

Certain groups may face problems in accessing needed services for other reasons [16]. Racial/ethnic disparities in older people’s use of acute and long-term care services are well documented [17, 18] and are believed to be attributable to factors that have implications both for different racial/ethnic groups’ predisposition to seek out services and for their access to services. However, evidence for racial/ethnic differences in home care services is mixed. Most studies show no statistically significant differences between African Americans’ and non-Hispanic whites’ receipt of most types of home care services after important predictors of services use have been taken into account [19, 20]. Among different racial/ethnic groups, evidence of underutilization of home care services seems to be strongest for Asian Americans [21, 22].

Disabled older people who are ineligible to receive home care services from public program sources or otherwise have insufficient access may use services financed by various personal sources such as out-of-pocket payment, private insurance, and family members. The few studies that have distinguished between who pays for home care services reveal the importance of out-of-pocket payment in financing these services [5, 6]. Using data from the NLTCS, Liu et al. [5] find that more than half of disabled older people who received home care services in 1982, 1989, and 1994 used out-of-pocket funds to pay for at least part of the services. However, evidence from this study as well as a study by Spector et al. [6] suggests that the importance of personal resources in paying for home care services declined during the early 1990s. Family members may also pay for services on behalf of elderly relatives.

While the role of the family in supporting elderly relatives through time and cash transfers is well documented [23, 24], little is known about the financial contributions of family members in paying for home care services. However, consistent with what Denton [25] refers to as “substitution model” regarding the relationship between older people’s informal and formal support systems more generally, we might expect that familial transfers to pay for services will be more common among those with difficulty in accessing publicly funded services.

We build on previous research on the financing of home care services by examining socio-demographic characteristics associated with the use of public program—namely, Medicare and Medicaid—and personal sources to pay for services. We also report results from supplementary analyses on services specifically financed by family members.

## Methods

### Sample

We use data from the National Long-Term Care Survey (NLTCS), which was conducted by faculty at the Center for Demographic Studies at Duke University. Secondary analysis of this data was approved by the School of Social and Political Science Research Office at the University of Edinburgh based on a level 1 assessment of ethical risks. The NLTCS sample is drawn from Medicare enrolment files covering at least 97 % of the U.S. community-dwelling and institutional elderly population at least 65 years old. A significant strength of the NLTCS is its renewing panel design in which respondents who were determined to be chronically disabled (i.e., for 90 or more days) on at least one of nine personal care tasks (activities of daily living [ADL’s]) and at least one of seven household management tasks (instrumental activities of daily living [IADL’s]), as well as a subsample of nondisabled respondents, are followed from the time they were screened into the study. Because the NLTCS has been repeatedly replenished during each wave with a supplementary sample of 65 to 69 year-olds to replace those who died between surveys, it continues to be representative of the entire elderly Medicare beneficiary population.

We focused on respondents who were chronically disabled and residing in the community at the time of the survey from the 1989, 1994, 1999, and 2004 waves of the NLTCS. After 10 % (*n* = 1033) of eligible respondents were dropped using listwise deletion, our analytical sample consisted of 8815 respondents. Respondents may have not participated in a subsequent detailed community survey following the wave in which they became disabled because they died during the 5 intervening years between surveys, they entered a nursing home or another long-term care institution, they could not be re-interviewed for other reasons, or regained full functioning and therefore were not asked the questions about caregivers. However, for 33 % of these respondents (*n* = 2910), we had information on their home care services use and the covariates for at least one additional wave. Thus, our analytical sample consisted of a total of 11,725 person-years. There were few differences between respondents who participated in multiple waves and a single wave on the variables we used in our models.

### Measures

#### Outcomes

Our models examine use of home care services provided by formal caregivers paid by (a) any source, (b) Medicare alone, (c) at least in part by Medicaid, (d) personal sources alone, and (e) both public (whether Medicare or Medicaid) and personal sources. Questions about paid caregivers are asked of NLTCS respondents who are determined to be chronically disabled and report that they received help to cope with a disability or health problem. Respondents are asked if the helper was paid and, if so, they are asked whether they themselves ended up paying any of the charges for the help out of their own pocket. In addition, respondents are asked about a number of other payment sources. Paying out of pocket, payment by private insurance, payment by an HMO, or payment by a family member (e.g., adult child) were categorized as personal sources.

#### Need-for-caregiving measures

The ADL’s and IADL’s that respondents were asked about on the detailed community survey differ somewhat from the functional limitations that were used to screen respondents into the study. On the detailed community survey respondents were asked about difficulty with performing 6 ADL’s and 8 IADL’s. The 6 ADL’s are eating, getting in or out of bed, getting around inside, dressing, bathing, and using the toilet. The 8 IADL’s are doing heavy work around the house (e.g., moving furniture), doing light work around the house (e.g., washing dishes), doing laundry, preparing meals, shopping for groceries, getting around outside, managing money, and taking medicine. In the NLTCS an individual is regarded as having difficulty in performing an ADL-related task if (1) they did not do the task under consideration, (2) received help in carrying out the task, or (3) used special equipment in carrying out the task. Difficulty with an IADL-related task is defined as not being able to perform the task because of a disability or health problem. To construct our measures of difficulty with ADL’s and IADL’s, we sum the number of tasks on which the respondents are considered to be disabled. In the analyses we treat number of ADL’s and IADL’s as categorical variables and distinguish between respondents having 0, 1 to 2, 3 to 4, and 5 to 6 ADL’s and 0 to 1, 2 to 3, 4 to 5, and 6 to 8 IADL’s. We do not distinguish between respondents with 0 and 1 IADL’s because of the small number of ADL-disabled respondents who did not report difficulty with any IADL-related task.

#### Other measures

Basic demographic characteristics of respondents that we included as covariates in our models are gender, Hispanic status, and a measure of race that distinguishes between Whites, African Americans, and respondents from other racial categories. We included family income, a measure of home assets, and educational attainment in our models as measures of respondents’ economic resources. The family income variable has five categories: “0–14,999,” “15,000–29,999,” “30,000–49,999,” “50,000–74,999,” and “75,000 and more” dollars per year. Membership in the lower two categories can be viewed as a “conservative” indicator of financial eligibility for Medicaid services. Nevertheless, because of very low income eligibility limits in many states, the lower two categories likely include at least some respondents who are ineligible for services based on financial and other criteria. The home assets variable captures information on both home ownership and home value among homeowners. It has three categories: “non-homeowners,” “owners of homes valued less than $150,000,” and “owners of homes valued more than $150,000.” Both family income and home assets are expressed in 2004 dollars. The educational attainment variable has three categories: “less than high school graduate,” “high school graduate,” and “college degree.” In addition to capturing non-housing wealth, this variable likely encompasses other dimensions such as health literacy and health status.

Measures of respondents’ informal caregiving resources that we included are hours of informal care that the respondent reported receiving during the previous week, marital status (four categories: single, married, widowed, divorced), and number of children. We include the latter two measures of informal caregiving resources to capture more subtle forms of help that family members may provide such as standby help or companionship.

Finally, we included year and state fixed effects in our models to capture change over time and differences across states in health policy, the organisation of the long-term care market, and population preferences [26–29].

We recode the continuous and count measures other than age into categorical variables to estimate possible nonlinear effects. It also facilitates treatment of missing values for family income and home assets. Table 1 presents the descriptive statistics for the covariates in our models.

**Table 1:** Description of analysis sample

	Percent	S.E.
*Need-for-caregiving measures*		
No. of ADL's		
0	34	(1.1)
1-2	42	(1.2)
3-4	16	(0.8)
5-6	8	(0.5)
No. of IADL's		
0-1	41	(1.2)
2-3	28	(1.0)
4-5	16	(0.8)
6-8	15	(0.8)
Age		
65–74	24	(0.9)
75–84	52	(1.0)
85+	24	(0.9)
*Demographic characteristics*		
Gender		
Female	43	(1.5)
Male	57	(1.5)
Race		
White	89	(0.9)
African American	10	(0.9)
Other	1	(0.3)
Hispanic		
No	95	(0.6)
Yes	5	(0.6)
Education		
Less than high school grad	53	(1.5)
High school grad	39	(1.4)
College degree	8	(0.7)
Family income		
0–14,999	37	(1.2)
15,000–29,999	27	(1.1)
30,000–49,999	9	(0.7)
50,000–74,999	3	(0.4)
75,000+	2	(0.3)
Family income missing	22	(0.9)
Home assets		
No	27	(1.2)
Yes, home value missing	32	(1.1)
Home value less than 150,000	32	(1.1)
Home value greater than 150,000	9	(0.6)
*Informal caregiving resources*		
Hours of informal care		
0	50	(1.2)
1–8	22	(0.9)
9–24	13	(0.8)
25+	15	(0.8)
Marital status		
Single	4	(0.4)
Married	40	(1.3)
Widowed	50	(1.3)
Divorced	6	(0.6)
Number of children		
0	15	(1.1)
1	16	(1.0)
2	23	(1.2)
3	19	(1.3)
4+	26	(1.3)

NLTCS, 1989–2004 (*N* = 11,725)

### Missing values

Cases with missing values on one or more covariates except for family income and home assets were dropped using listwise deletion, because missing values on these variables were not a serious problem. However, 23 % of the cases had missing values on family income and 30 % of the cases had missing values on home value. Rather than completely remove these observations from the analysis, we created an additional category for each of the categorical covariates based on these variables, and person-years with missing information on that covariate were assigned to that category (*not* the reference category). For instance, for family income the reference category is “income less than $15,000,” and the 6th category indicates that the income information is missing. Thus, for example, in the hours of care equations the coefficient for the missing category indicates average hours of care received for those with missing income information relative to the reference category. The advantages of this procedure for dealing with missing values are that it allows us to retain as much information on these observations as possible and to examine whether person-years with missing information on a covariate are statistically significantly different with regard to home care services use than cases with non-missing values [30]. As a robustness check of our findings, we also performed multiple imputations for family income and home assets using chained equations [31]. The point estimates and standard errors for family income, home assets, and the other variables based on the multiple imputations were very similar to the original estimates (Additional files 1 and 2).

### Analytical strategy

We examine use of home care services financed by different funding sources. A limitation not unique to the NLTCS data is that for those receiving care from a single caregiver paid by more than one source, it is not possible to divide up hours of care received based on the funding source. Thus, hours of care received from these caregivers were placed in a separate category (i.e., “both public and personal sources”). By contrast, we were able to divide up care received by funding source for those receiving care provided by *multiple* paid caregivers.

Our analysis is split into two parts. We first examine socio-demographic characteristics associated with the odds of receiving any services from a source. We then examine factors associated with the number of hours conditional on receiving at least one hour from that source. Because the distribution of hours of care received from each source is skewed to the right, we take the natural log of these variables (see Additional file 3 for descriptive statistics for the dependent variables used in the models).

While we control for a number of important socio-demographic characteristics that affect use of home care services (Table 1) in each part of the analysis, it is still possible that there are some omitted persistent influences on their use. For instance, these might reflect unmeasured aspects of a person’s friendship and kin network, or wealth, which affect their use of formal sources of care. In studying the variation in paid home care services use among people and over time, we assume that these omitted factors are not correlated with the other explanatory variables. Thus, we exploit the panel nature of the data and employ “random effects” models to obtain more efficient (precise) estimates of the effects of both time-varying and time-invariant characteristics. Variables that we treat as time-varying, or for which there is substantial within-person variation in the NLTCS, are hours of informal care, number of ADL’s and IADL’s, age, family income, home assets, and marital status, while time-invariant variables are gender, racial ancestry, educational attainment, and number of children. The random effect estimates of the time-varying characteristics on our outcomes represent a weighted average of the “effects” of the within-person and between-person variation. We use “seemingly unrelated estimation” to take into account that equations for the four sources of paid care may be interdependent and are estimated on the same sample (giving rise to cross-equation covariance in the error terms), and we compute robust (clustered sandwich) standard errors, which takes into account correlation of the error terms across time for the same individual.

We condition on hours of informal care because it may be driven mainly by the availability of family in close proximity to provide such care. But this variable, ADL’s, IADL’s, and income are likely to be endogenous with respect to paid home care services; that is, both person and time components of the equation error terms may be correlated with these variables. Accordingly, we should interpret the estimated parameters as associations rather than effects of exogenous variation in these variables.

“Fixed effects” estimates (to use the language of panel data analysis) make a weaker assumption about the omitted persistent influences. These allow for unobserved heterogeneity in respondents’ time-invariant characteristics to be correlated with the explanatory variables. They are based entirely on within-individual variation in the time-varying characteristics [32]. In the analysis here, fixed effect estimates of the ‘impacts’ of the observed covariates are similar to the random effect estimates. Nevertheless, fixed effect estimates still do not eliminate the likely danger of endogeneity, because of correlation between the time component of the error term and the explanatory variables.

## Results and discussion

### Percentage of caregivers and hours of care given by payer

The 2428 older people (21 %) receiving formal care in our sample identified 3436 paid caregivers, which represents an average of 1.42 paid caregivers per care recipient. Table 2 shows the percentage of caregivers paid by each funding source and hours of care given by funding source. Personal sources represent the most important funding source for home care services. 63 % of caregivers are paid by personal sources alone and provide 62 % (10.1 h) of the total hours of home care services. A further 9 % of caregivers receive payment from both public program and personal sources. By contrast, 28 % of caregivers are paid by publicly funded programs alone and provide only about one-quarter of home care services (24 %, 3.9 h). Table 2 excludes the 102 caregivers solely paid by an “other” source.

**Table 2:** Percentage of caregivers and hours of home care given by payer

Funding source	Percentage of caregivers	Hours of care given	
		Number	Percent
All funding sources	100	16.2	100
Publicly funded programs	28	3.9	24
Personal sources	63	10.1	62
Both public and personal sources	9	2.2	13
Publicly funded programs only	100	12.1	100
Medicare	60	5.4	45
Medicaid	22	2.9	24
Both	18	3.9	32
Personal sources only	100	17.7	100
Out-of-pocket payment	87	13.2	75
Private insurance	4	1.3	7
Family member	2	1.0	6
>1 personal source	8	2.1	12
Both public and personal sources	100	21.4	100

NLTCS, 1989–2004 (*N* = 3436 caregivers)

Six in ten publicly financed caregivers are paid by Medicare alone. However, these caregivers provide less than half of publicly financed services (45 %, 5.4 h). 22 % of publicly financed caregivers are paid by Medicaid alone and provide about one-quarter of the services (24 %, 2.9 h). Finally, 18 % of these caregivers receive payment from both Medicare and Medicaid and provide about one-third of the services (32 %, 3.9 h).

Out-of-pocket payment is easily the most prevalent personal funding source for home care services. Almost 9 in 10 personally financed caregivers (87 %) are paid out-of-pocket and provide three-quarters of personally financed services (13.2 h). By contrast, fewer than 5 % of these caregivers are paid by either private insurance (4 %) or a family member of the older person (2 %). Finally, 8 % of personally financed caregivers receive payment from more than one personal source and provide 12 % of personally financed services (2.1 h).

### Characteristics related to receipt of care

Overall, the odds of receiving at least one hour of home care services (regardless of payer) are about two times greater (1.98, *p* < .01) for older people with family incomes of over 75,000 dollars per year compared to the odds for those with family incomes less than 15,000 dollars per year (Table 3). The positive relationship between family income and overall services use is explained by economic disparities in receipt of care from personal sources alone (Table 4, col. C). However, economic disparities in overall services use would be even greater in the absence of government provision. Even after controlling for characteristics that capture older people’s need for services, the odds of receiving services financed at least in part by Medicaid for older people with family incomes of over 30,000 dollars per year are 0.22 times (*p* < .01) the odds for those in the lowest income category (Table 4, col. B). The odds ratios representing differences by family income in the odds of receiving services financed by Medicare alone are all less than one (Table 4, col. A). However, only the odds ratios for the second (15,000–29,999 dollars per year) and third income categories (30,000–49,999 dollars per year) are statistically significant (*p* < .05 and *p* < .01, respectively). Finally, the odds ratios representing differences by family income in the odds of receiving services jointly financed by public program and personal sources are all less than one and statistically significant at conventional levels (Table 4, col. D).

**Table 3:** Odds ratios from logistic regression model explaining home care receipt

	Odds ratio	(95 % CI)
*Need for caregiving*		
No. of ADL’s		
1-2	1.11	(0.96–1.29)
3-4	1.85**	(1.57–2.17)
5-6	3.19**	(2.61–3.89)
No. of IADL's		
2-3	2.40**	(2.03–2.82)
4-5	4.27**	(3.56–5.12)
6-8	6.00**	(4.91–7.33)
Age	1.02**	(1.01–1.03)
*Demographics*		
Female	0.95	(0.85–1.07)
Race		
African American	0.68**	(0.56–0.83)
Other	0.80	(0.45–1.41)
Hispanic	1.00	(0.76–1.32)
*Economic resources*		
Education		
High school grad	1.45**	(1.29–1.64)
College degree	2.22**	(1.83–2.71)
Family income		
15,000–29,999	1.07	(0.92–1.23)
30,000–49,999	1.06	(0.84–1.34)
50,000–74,999	1.15	(0.86–1.54)
75,000+	1.98**	(1.33–2.95)
Missing	0.93	(0.81–1.06)
Home assets		
Yes, but missing	0.65**	(0.56–0.74)
<150,000	0.62**	(0.54–0.71)
≥150,000	0.66**	(0.54–0.80)
*Year*		
1994	1.13	(0.98–1.29)
1999	0.59**	(0.50–0.69)
2004	0.36**	(0.31–0.43)
*Informal resources*		
Informal care hours		
0	4.37**	(3.66–5.21)
1-8	2.44**	(2.05–2.91)
9-24	1.35**	(1.12–1.62)
Marital status		
Single	1.06	(0.80–1.42)
Widowed	1.47**	(1.28–1.68)
Divorced	1.49**	(1.21–1.83)
Number of children		
1	0.71**	(0.59–0.85)
2	0.69**	(0.58–0.81)
3	0.63**	(0.52–0.76)
4+	0.61**	(0.51–0.73)

NLTCS, 1989-2004 (N = 11.725). Model includes state fixed effects

*p < .05, **p < .01

**Table 4:** Odds ratios from logistic regression models explaining home care receipt by payer

	Public program only							
	Medicare only [A]		Medicaid [B]		Personal only [C]		Public & Personal [D]	
	Odds ratio	(95 % CI)	Odds ratio	(95 % CI)	Odds ratio	(95 % CI)	Odds ratio	(95 % CI)
*Need for caregiving*								
No. of ADL's								
1-2	2.53**	(1.49–4.32)	1.42	(0.88–2.27)	0.96	(0.82–1.13)	1.55	(0.95–2.52)
3-4	4.79**	(2.81–8.16)	2.74**	(1.72–4.38)	1.34**	(1.12–1.62)	2.78**	(1.65–4.71)
5-6	6.10**	(3.45–10.78)	3.37**	(1.98–5.72)	2.00**	(1.59–2.51)	6.54**	(3.77–11.33)
No. of IADL's								
2-3	2.47**	(1.41–4.34)	2.32**	(1.26–4.28)	2.28**	(1.91–2.72)	2.19**	(1.24–3.85)
4-5	4.68**	(2.66–8.22)	5.71**	(3.12–10.46)	3.55**	(2.89–4.35)	3.39**	(1.83–6.28)
6-8	6.17**	(3.44–11.06)	9.66**	(5.20–17.97)	4.27**	(3.40–5.36)	4.38**	(2.33–8.24)
Age	0.99	(0.98–1.01)	0.98*	(0.96–1.00)	1.03**	(1.02–1.04)	1.02	(1.00–1.03)
*Demographics*								
Female	0.88	(0.68–1.14)	0.91	(0.68–1.22)	0.92	(0.80–1.05)	1.22	(0.94–1.58)
Race								
African American	1.02	(0.70–1.49)	1.56*	(1.10–2.22)	0.44**	(0.33–0.58)	0.72	(0.50–1.06)
Other	1.05	(0.38–2.91)	0.80	(0.30–2.14)	0.96	(0.49–1.88)	0.27	(0.03–2.20)
Hispanic	0.67	(0.37–1.21)	1.23	(0.75–2.03)	1.02	(0.74–1.41)	0.75	(0.42–1.36)
*Economic resources*								
Education								
High school grad	1.01	(0.77–1.33)	0.56**	(0.41–0.78)	1.79**	(1.56–2.06)	1.10	(0.84–1.44)
College degree	0.96	(0.59–1.57)	0.12**	(0.04–0.39)	2.90**	(2.37–3.56)	1.89**	(1.25–2.86)
Family income								
15,000-29,999	0.67*	(0.49–0.92)	0.28**	(0.18–0.44)	1.72**	(1.46–2.04)	0.63**	(0.46–0.87)
30,000-49,999^**a**^	0.40**	(0.21–0.77)	0.22**	(0.09–0.57)	1.79**	(1.39–2.31)	0.48*	(0.26–0.89)
50,000-74,999	0.48	(0.22–1.05)	(variable omitted)^**a**^		2.13**	(1.56–2.91)	0.45*	(0.20–0.99)
75,000+	0.37	(0.09–1.57)	(variable omitted)^**a**^		3.72**	(2.50–5.53)	0.12*	(0.02–0.92)
Missing	0.52**	(0.38–0.72)	(0.26–0.54)		1.41**	(1.20–1.65)	0.82	(0.61–1.10)
*Economic resources*								
Home assets								
Yes, but missing	0.87	(0.64–1.17)	0.52**	(0.37–0.73)	0.74**	(0.63–0.87)	0.68*	(0.50–0.93)
< 150,000	0.95	(0.70–1.31)	0.43**	(0.31–0.61)	0.72**	(0.62–0.85)	0.52**	(0.39–0.70)
≥ 150,000	0.70	(0.41–1.20)	0.14**	(0.05–0.40)	0.86	(0.70–1.07)	0.60*	(0.39–0.93)
*Year*								
1994	3.48**	(2.47–4.89)	1.06	(0.74–1.53)	1.12	(0.96–1.31)	0.32**	(0.24–0.45)
1999	1.99**	(1.33–2.97)	1.14	(0.78–1.67)	0.49**	(0.41–0.59)	0.39**	(0.28–0.55)
2004	0.89	(0.57–1.40)	1.09	(0.75–1.58)	0.34**	(0.28–0.40)	0.15**	(0.10–0.23)
*Informal resources*								
Informal care hours								
0	1.28	(0.92–1.79)	3.84**	(2.56–5.74)	5.17**	(4.18–6.39)	1.20	(0.85–1.71)
1-8	1.18	(0.83–1.67)	2.31**	(1.51–3.52)	2.98**	(2.40–3.70)	1.42*	(1.01–2.00)
9-24	1.00	(0.70–1.42)	1.20	(0.75–1.93)	1.63**	(1.29–2.06)	1.00	(0.70–1.43)
Marital status								
Single	0.55	(0.29–1.05)	1.09	(0.56–2.13)	1.34	(0.97–1.85)	0.89	(0.49–1.61)
Widowed	0.91	(0.67–1.22)	1.27	(0.86–1.89)	1.65**	(1.41–1.93)	1.03	(0.76–1.39)
Divorced	1.13	(0.71–1.78)	1.83*	(1.14–2.94)	1.49**	(1.16–1.92)	0.82	(0.46–1.46)
Number of children								
1	0.51**	(0.34–0.76)	0.59*	(0.38–0.94)	0.81*	(0.67–0.99)	0.88	(0.60–1.30)
2	0.52**	(0.36–0.76)	0.54**	(0.35–0.84)	0.86	(0.71–1.04)	0.57**	(0.38–0.84)
3	0.52**	(0.35–0.79)	0.73	(0.45–1.19)	0.70**	(0.57–0.86)	0.73	(0.49–1.11)
4+	0.57**	(0.40–0.81)	0.95	(0.64–1.42)	0.61**	(0.49–0.75)	0.70	(0.48–1.03)

NLTCS, 1989–2004 (*N* = 11,725). Models include state fixed effects

*p < .05, **p < .01

^a^Family income was topcoded at $30,000+ in the Medicaid model due to the very small number of respondents with incomes over $30,000 receiving services financed by Medicaid. Therefore, the second income dummy variable gives the difference in the outcome between those with incomes over $30,000 and those in the lowest income category

We included education in the models as a rough indicator of respondents’ non-housing wealth, for which the NLTCS does not have direct measures. Similar to the results for family income, there is a strong positive relationship between respondents’ educational attainment and the odds of using personally financed services. The odds for high school graduates are 1.79 times greater compared to the reference category (*p* < .01), while the odds for college graduates are 2.90 times greater (*p* < .01) (Table 4, col. C).

With regard to respondents’ home assets, we found that, contrary to our expectations, the odds of using personally financed services are statistically significantly lower for owners of homes valued at less than 150,000 dollars compared to non-homeowners (*p* < .01) (Table 4, col. C). Also, contrary to our expectations, the odds of using services jointly financed by public program and personal sources are statistically significantly lower among homeowners, regardless of reported home value information (*p* < .01) (Table 4, col. D). We suspect that these counterintuitive findings reflect the possibility of greater liquid assets of non-homeowners. Indeed, a major reason for selling a home identified by respondents in the NLTCS is “to help cover medical expenses.”

In line with our expectations, the odds of receiving services financed at least in part by Medicaid are statistically significantly lower for homeowners, regardless of reported home value information (*p* < .01). Furthermore, this “effect” appears to be larger for owners of homes worth at least 150,000 dollars (Table 4, col. B). The fact that there are any recipients at all of Medicaid-financed services among this group of homeowners may be due to the exclusion of owner-occupied housing from some states’ definitions of countable assets [13]. By contrast, differences by respondents’ home assets in the odds of receiving services financed by Medicare were not statistically significant (Table 4, col A).

Our results suggest that minority racial/ethnic groups are not disadvantaged in terms of their access to publicly funded home care services. In fact, African Americans’ odds of receiving services financed at least in part by Medicaid is 1.56 times greater than Whites’ odds (*p* < .05) (Table 4, col. B). However, we did find that African Americans’ odds of receiving personally financed services is 0.44 times lower than Whites’ odds (*p* < .01) even after controlling for a number of socio-demographic characteristics in our model (Table 4, col. C). Interestingly, in contrast to previous research on gender disparities in receipt of home care services (see, e.g., [33]), we find no statistically significant differences by gender in the odds of overall services use (Table 3) and services use by funding source (Table 4).

With regard to the need-for-caregiving measures, even after controlling for number of ADL’s and IADL’s, age is positively related to older people’s overall services use. The odds of services use increases by a factor of 1.02 for each 1-year increase in age (*p* < .01) (Table 3). Furthermore, the relative importance of different payers changes with respondents’ age. The odds of using personally financed services are 1.03 times larger for each 1-year increase in age (*p* < .01) (Table 4, col. C), while the odds of using services financed at least in part by Medicaid are 0.98 times smaller for each 1-year increase in age (*p* < .05) (Table 4, col. B).

Older people with difficulty in performing a larger number of ADL’s and IADL’s are substantially more likely to receive home care services overall than less impaired older people. The odds of receiving services is 3.19 times greater for older people with 5-6 ADL’s compared to those with no ADL’s (*p* < .01). Older people with 6-8 IADL’s, furthermore, have a 6.00 times greater odds of receiving services overall than older people with 0-1 IADL’s (*p* < .01) (Table 3). With regard to the average marginal effects, the probability of receiving services from any source is .17 greater for older people with 5-6 ADL’s compared to those with no ADL’s, on average, and .24 greater for older people with 6-8 IADL’s compared to those with 0-1 IADL’s.

While more impaired older people are more likely to receive services across payers, our results suggest that the relative importance of public program sources in financing services increases substantially with number of ADL’s and IADL’s. For example, the odds of receiving services financed by Medicare alone are greater by a factor of about 6 for those with 5-6 ADL’s (6.10, *p* < .01) and those with 6-8 IADL’s (6.17, *p* < .01) (Table 4, col. A). The odds of using services financed at least in part by Medicaid are 3.37 times greater for older people with 5-6 ADL’s (*p* < .01) and 9.66 times greater for older people with 6-8 IADL’s (*p* < .01) (Table 4, col. B).

Older people with fewer informal caregiving resources, whether captured as hours of informal care received, marital status, or number of children, are also substantially more likely to use home care services overall. The odds of using paid services are 4.37 times greater for older people who reported not receiving any informal care during the past week compared to those who received more than 24 h of informal care (*p* < .05) (Table 3). With regard to the average marginal effects, the probability of receiving services is .19 greater for older people who did not receive any informal care. The relationship between hours of informal care received and overall services use appears to be primarily due to differences in older people’s use of services paid for by personal sources alone (Table 4, col. C) and services paid for at least in part by Medicaid (Table 4, col. B).

The odds of using services overall are also about 1.5 times greater among the widowed (1.47, *p* < .01) and divorced (1.49, *p* < .01) compared to married older people. The odds of using services are substantially lower for parents regardless of number of children (*p* < .01) (Table 3). These latter two findings likely reflect the importance of more subtle forms of help provided by spouses and adult children, respectively, such as standby help or companionship.

Finally, the odds of receiving services overall were substantially lower in 1999 (0.59, *p* < .01) and 2004 (0.36, *p* < 0.01), after controlling for the socio-demographic characteristics in our model, compared to 1989 (Table 3). These odds ratios correspond to average marginal effects on the probability of receipt of −.07 for 1999 and −.13 for 2004. This trend is primarily due to a decrease over time in service receipt from personal sources alone. The odds ratio for 2004 compared to 1989 (0.34, *p* < .01) corresponds to an average marginal effect of −.10 (Table 4, col. C). In addition, there has been a decrease over time in the odds of receiving services paid for by Medicare alone (compared to 1994) and a combination of public program and personal sources (Table 4). By contrast, there was no statistically significant change in the odds of receiving services financed at least in part by Medicaid (Table 4, col. B).

### Characteristics related to log hours of care

Characteristics associated with the log hours of care include number of ADL’s and IADL’s, age, racial ancestry, family income, year, hours of informal care, marital status, and number of children. Again, we did not find evidence of any minority disadvantage in access to publicly funded home care services. In fact, African Americans receive 84 % (100 ∗ *β*) more hours of care and Hispanics receive 104 % more hours of care financed by Medicare alone than Whites (*p* < .01) (Table 5, col. B). Older people with difficulty in performing 5-6 ADL’s receive 68 % more hours of care financed by Medicare alone (*p* < .05), 80 % more hours financed at least in part by Medicaid (*p* < .05), and 104 % more hours financed by personal sources alone (*p* < .01). We also find additional evidence that older people with fewer informal caregiving resources are more reliant on paid home care services. Older people who reported not receiving any informal care during the past week used 55 % more hours of services paid for by Medicare alone (*p* < .05), 60 % more hours paid for at least in part by Medicaid (*p* < .05), and 57 % more hours paid for by personal sources alone (*p* < .01). Finally, we find that older people with family incomes of at least 75,000 dollars per year use 74 % more hours of personally financed services than older people in the lowest income category (*p* < .01). However, none of the other coefficients for family income from this model are statistically significant.

**Table 5:** OLS regression models explaining log hours of home care by payer

	All sources [A]		Public program only				Personal only [D]		Public & Personal [E]	
			Medicare only [B]		Medicaid [C]					
	Coef.	S.E.	Coef.	S.E.	Coef.	S.E.	Coef.	S.E.	Coef.	S.E.
*Need for caregiving*										
No. of ADL's										
1-2	−.01	(.08)	.50	(.29)	.60*	(.27)	−.06	(.09)	−.72	(.37)
3-4	.34**	(.09)	.46	(.30)	.78**	(.29)	.22*	(.10)	−.22	(.38)
5-6	.92**	(.10)	.68*	(.30)	.80*	(.32)	1.04**	(.12)	.28	(.41)
No. of IADL's										
2-3	.24**	(.09)	−.23	(.37)	.04	(.43)	.27**	(.10)	.21	(.41)
4-5	.56**	(.10)	.06	(.36)	.07	(.46)	.49**	(.11)	.57	(.36)
6-8	1.12**	(.11)	.21	(.37)	.54	(.48)	1.22**	(.13)	.76*	(.35)
Age	.01	(.00)	−.01	(.01)	.01	(.01)	.01**	(.00)	.00	(.01)
*Demographics*										
Female	.04	(.06)	−.08	(.16)	.13	(.17)	.03	(.07)	.06	(.21)
Race										
African American	.06	(.10)	.84**	(.23)	.05	(.21)	−.26	(.16)	.09	(.31)
Other	−.28	(.36)	−.80	(.59)	.05	(.41)	−.09	(.39)	−1.15	(.87)
Hispanic	.44**	(.13)	1.04**	(.38)	.00	(.29)	.42*	(.17)	.54	(.47)
*Economic resources*										
Education										
High school grad	.02	(.06)	.04	(.16)	.09	(.18)	.03	(.08)	−.22	(.22)
College degree	.11	(.09)	.00	(.45)	.29	(.91)	.13	(.10)	−.28	(.33)
Family income										
15,000–29,999	.02	(.08)	−.04	(.22)	.33	(.31)	.05	(.09)	.11	(.27)
30,000–49,999^a^	.12	(.12)	.19	(.44)	−.69	(.89)	.09	(.13)	.29	(.61)
50,000–74,999	.09	(.16)	.17	(.66)	(var omitted)^a^		−.05	(.17)	.82	(.55)
75,000+	.69**	(.17)	−.51	(.48)	(var omitted)^a^		.74**	(.17)	−.25	(.46)
Missing	.23**	(.08)	.18	(.21)	.16	(.20)	.23*	(.10)	.46*	(.23)
*Economic resources*										
Home assets										
Yes, but missing	−.01	(.08)	.03	(.18)	−.12	(.18)	.14	(.10)	−.25	(.29)
< 150,000	−.02	(.07)	−.09	(.18)	−.13	(.19)	−.02	(.09)	−.07	(.23)
≥ 150,000	.12	(.11)	.03	(.38)	−.47	(.42)	.21	(.12)	−.15	(.37)
*Year*										
1994	.01	(.07)	.09	(.19)	−.08	(.19)	−.11	(.09)	.47	(.25)
1999	−.09	(.08)	−.06	(.23)	−.25	(.21)	−.17	(.10)	.41	(.32)
2004	−.16	(.08)	.26	(.28)	−.36	(.21)	−.30**	(.10)	.49	(.37)
*Informal resources*										
Informal care hours										
9-24	.36**	(.11)	.12	(.22)	−.01	(.27)	.33*	(.15)	.16	(.31)
1-8	.55**	(.10)	.41	(.24)	.13	(.24)	.38**	(.13)	.27	(.31)
0	.73**	(.10)	.55*	(.23)	.60*	(.24)	.57**	(.13)	.52	(.31)
Marital status										
Single	.05	(.13)	.14	(.28)	.37	(.40)	−.03	(.16)	−.05	(.47)
Widowed	.19**	(.07)	−.05	(.21)	.21	(.25)	.22*	(.09)	.20	(.25)
Divorced	.08	(.11)	.09	(.30)	−.02	(.30)	−.03	(.14)	.08	(.47)
Number of children										
1	−.10	(.09)	.48	(.28)	.00	(.29)	−.18	(.11)	.15	(.29)
2	−.05	(.08)	.19	(.21)	.60*	(.26)	−.15	(.10)	−.15	(.28)
3	.00	(.09)	.44	(.27)	.02	(.24)	−.08	(.12)	.26	(.30)
4+	−.04	(.09)	.43	(.24)	.04	(.20)	−.19	(.12)	.27	(.30)

NLTCS, 1989–2004 (all sources model *N* = 2428, Medicare only *N* = 335, Medicaid *N* = 313, personal only *N* = 1621, public & personal *N* = 326). Models include state fixed effects

*p < .05, **p < .01

^a^Family income was topcoded at $30,000+ in the Medicaid model due to the very small number of respondents with incomes over $30,000 receiving services financed by Medicaid. Therefore, the second income dummy variable gives the difference in the outcome between those with incomes over $30,000 and those in the lowest income category

**Fig. 1:**
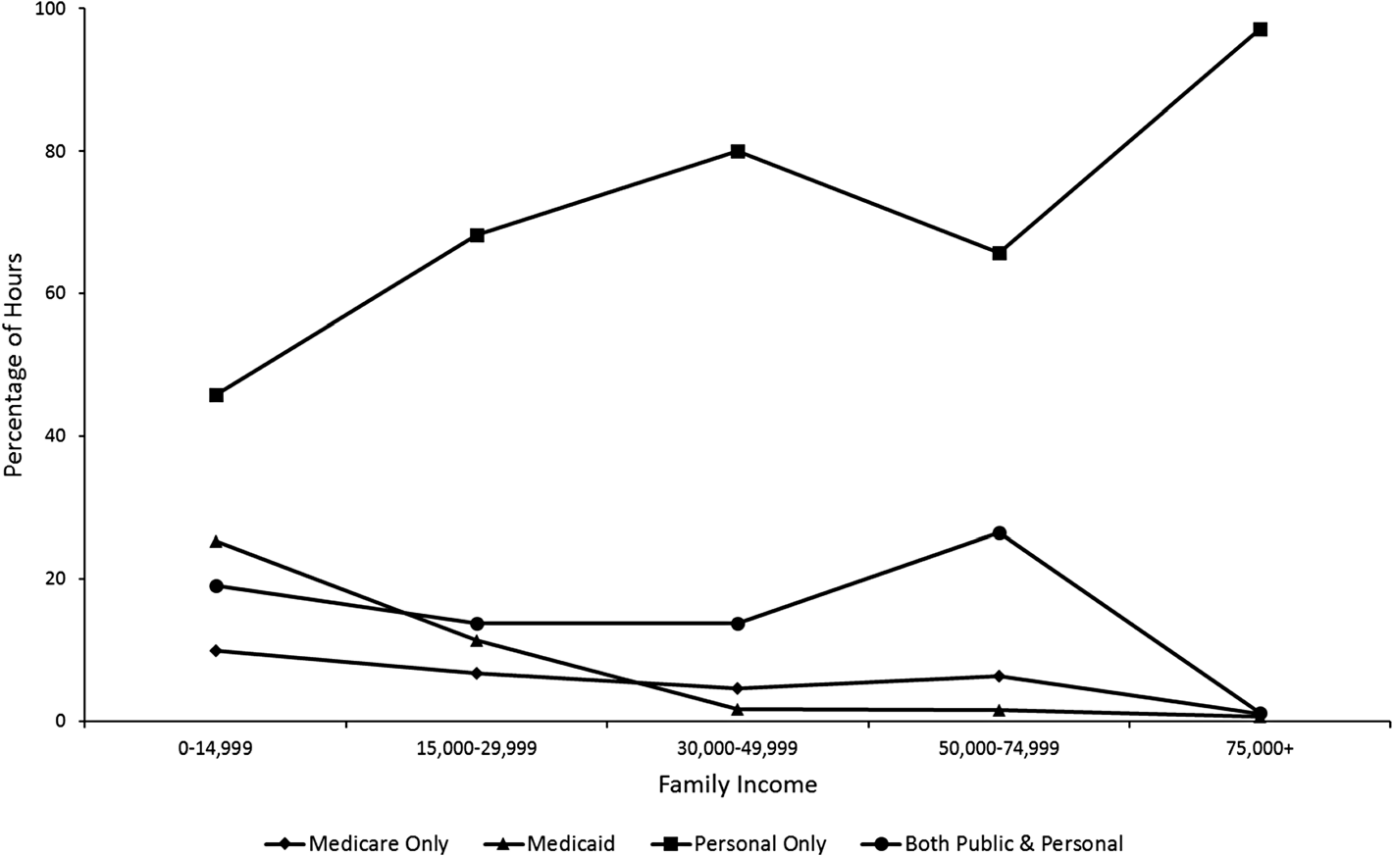
Percentage of hours received from each payer by family income

**Fig. 2:**
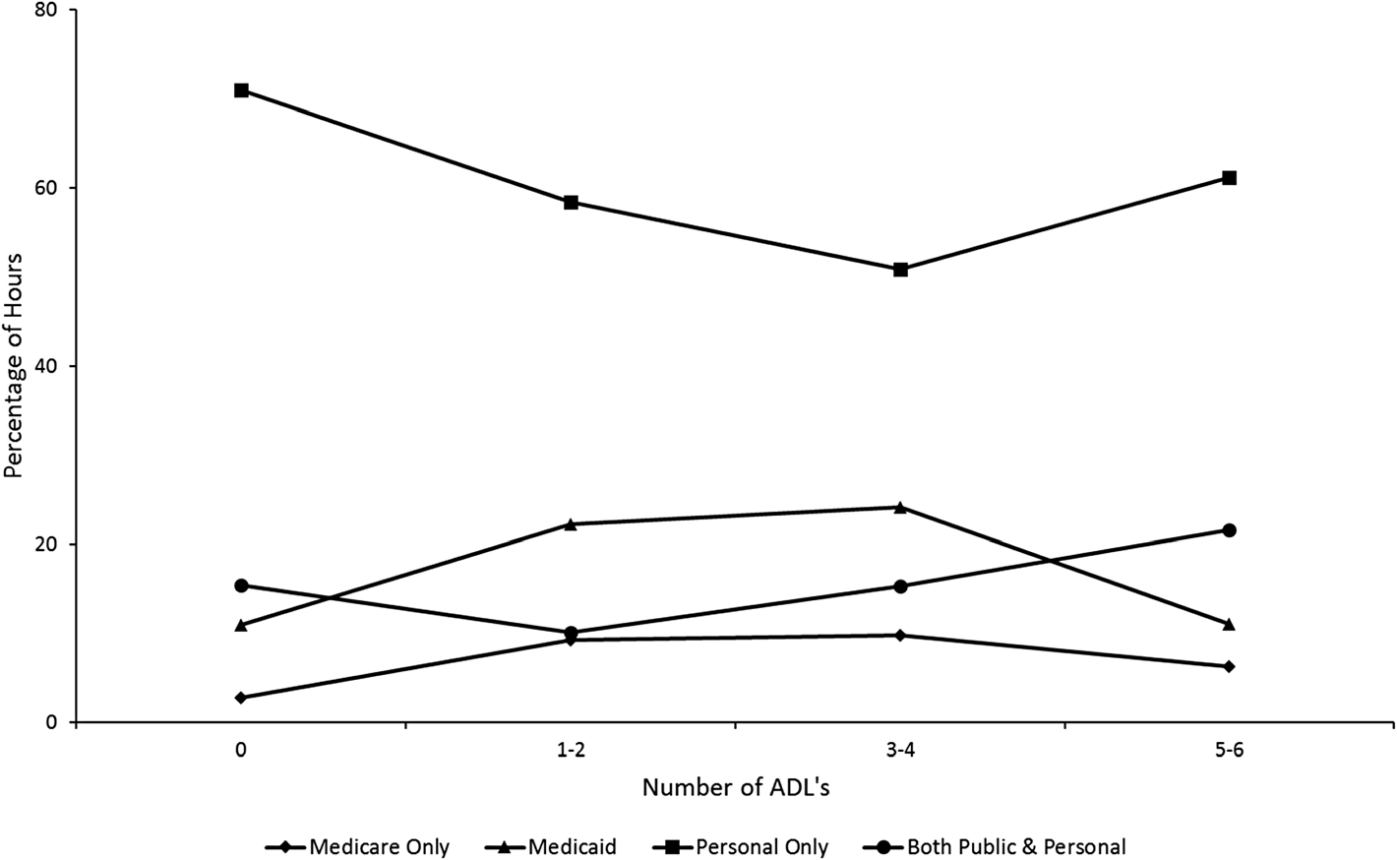
Percentage of hours received from each payer by number of ADL's

Figures 1 and 2 give the percentage of care hours received from each payer by family income and number of ADL’s, respectively. The percentages in these figures are based on the combined prediction of caregiving hours from the models explaining care receipt and log hours of care (see Additional file 4 for predicted hours by family income and Additional file 5 for predicted hours by number of ADL’s). The combined prediction is the product of the probability of receiving at least one hour of home care services from the first part of the analysis and the expected value of caregiving hours from the second part. These figures show more clearly than the model coefficients how the funding mix for home care services differs by family income and number of ADL’s.

Older people in every income category except for the lowest income category receive at least 60 % of services from caregivers paid by personal sources alone (i.e., out-of-pocket payment, private insurance, or a family member). Among older people with family incomes of less than 15,000 dollars per year, the funding mix is more evenly split between public program and personal sources (Fig. 1). Figure 2 shows a decrease in the relative importance of personal sources with a rise in the number of ADL’s. However, personal sources again become somewhat more important among older people with 5-6 ADL’s.

### Supplementary analyses

We also estimated logistic regression models explaining receipt of care financed by Medicaid alone (Additional file 6) and family members (Additional file 7). Results from the former model were similar to results from the model, previously discussed, that includes “dual eligibles” for Medicare and Medicaid. Furthermore, we found that characteristics associated with receipt of services paid for by family members and any personal source are similar. However, in the model explaining receipt of services paid for by family members, none of the variables capturing informal caregiving resources were statistically significant.

## Conclusions

Home care services are both a valuable and common source of care for older people across countries. However, because of the significant cost of these services, understanding the ability of those who are not covered by relevant public programs to access services financed by other means is essential to evaluating the success of policy in promoting equitable access. While the funding mix for older people’s general health care costs has received attention from previous researchers [34, 35], few studies have focused specifically on the relative importance of public program and personal sources in financing home care services. We consider this question using nationally representative data on disabled older Americans.

Given our focus on different payers for home care services, the NLTCS has unique strengths as a data set. However, readers should be aware of two limitations of the NLTCS data. First, because the last survey was fielded in 2005, our results do not reflect recent developments in publicly funded HCBS such as the expansion of Medicaid under the Affordable Care Act [36]. For this reason current economic disparities in receipt of services might be less severe. Second, respondents with health problems are not asked the questions about paid caregivers in the NLTCS unless they report a chronic disability. Thus, we likely underestimate use of skilled home health care. Keeping these limitations in mind, we have three principal findings.

First, this study shows that older people with family incomes over 75,000 dollars per year receive 8.5 more hours of home care overall than those making less than 15,000 dollars per year (Additional file 4). The economic disparities are explained by higher income people’s greater use of personally financed services. In fact, almost all (97 %) home care received by those with family incomes over 75,000 dollars per year are financed by personal sources alone. Therefore, our results suggest that higher income people’s use of these services is more than sufficient to compensate, at least in terms of hours of services received, for their low usage rates of services from public program sources.

However, a significant caveat is that our findings do not address likely differences between payers in provider type and type of care given. For example, most personally financed services are provided by self-employed, independent providers, which involve trade-offs in favour of lower rates and greater consumer control [7]. Therefore, services financed by different payers should not necessarily be viewed as complete substitutes.

Second, while Medicaid is the most important payer for long-term (as opposed to post-acute) home care services in the United States, its reach is rather limited in two ways. First, the large majority of respondents in our sample who reported receiving services financed by Medicaid had family incomes of less than 15,000 dollars per year. Second, even among this group, 65 % of services are provided by caregivers paid in whole or in part from personal sources. Nevertheless, economic disparities in home care services use would be even greater in the absence of public provision. Back-of-the-envelope calculations indicate that if users of services financed by public program sources alone no longer received such services, the disparity in home care services use between older people in the lowest and highest income categories would be 21 % greater (10.3 h compared to 8.5 h).

Third, consistent with previous research, our results reveal substantial differences in older people’s services use according to “need,” whether captured by older people’s functional limitations or informal caregiving resources. The substantial differences in services use by hours of informal care received is consistent with arguments that Cantor [37] makes in explicating a “hierarchical compensatory theory of social supports.” According to this theory, older people only turn to formal support, such as paid home care services, when the informal support network is unavailable or overburdened.

In addition, our results suggest that older people with “moderate” levels of impairment (e.g., 1-2 or 3-4 ADL’s) disproportionately rely on services financed by public programs. However, personal sources again become somewhat more important among older people with 5-6 ADL’s. These findings may point to the dual influence of functional need eligibility criteria in restricting access to services for less impaired older people and service limits in restricting access to services among the most impaired group.

In summary, the reach of public programs that pay for home care services is restricted by both the size of the eligible population and limits on services. As a result, even older people with modest economic resources rely heavily on personally financed services. This has implications for both disabled older people and their families. Expenditures on home care services and other health care services can easily threaten older people’s economic well-being [38]. The depletion of older people’s economic resources has implications for children’s well-being and the reproduction of economic inequality in the next generation through smaller inter vivos transfers and bequests [39]. Finally, if informal caregivers step in when paid services are inadequate, they bear significant economic and noneconomic costs (see Fast et al. [3] for an excellent taxonomy of these costs). Therefore, in interpreting these results, it is important to recognize that who pays for home care services has broad implications. From such a perspective, long-term care policy is not just health policy but family policy.

## Additional files

Additional file 1:
**Odds Ratios From Logistic Regression Models Explaining Home Care Receipt by Payer (With Multiple Imputations (5) for Family Income and Home Assets).** (PDF 81 kb) Additional file 2:
**OLS Regression Models Explaining Log Hours of Home Care by Payer (With Multiple Imputations (5) for Family Income and Home Assets).** (PDF 81 kb) Additional file 3:
**Descriptive Statistics for Home Care Services Use by Payer.** (PDF 47 kb) Additional file 4:
**Care Hours Received From Each Payer by Family Income.** (PDF 24 kb) Additional file 5:
**Care Hours Received From Each Payer by Number of ADL’s.** (PDF 24 kb) Additional file 6:
**Odds Ratios From Logistic Regression Models Explaining Receipt of Care Provided by Caregivers Paid by Medicaid Alone.** (PDF 76 kb) Additional file 7:
**Odds Ratios From Logistic Regression Models Explaining Receipt of Care Provided by Caregivers Paid by Family Members.** (PDF 76 kb)

## Abbreviations

NLTCS: National long-term care survey
HCBS: Home and community based services
