# Supplementary material for: Who pays for home care? A study of nationally representative data on disabled older Americans
Source: BMC Health Serv Res. 2015 Jul 31;15:301. doi: 10.1186/s12913-015-0978-x (PMC4521465; doi:10.1186/s12913-015-0978-x)
Supplement: Additional file 1: — Odds Ratios From Logistic Regression Models Explaining Home Care Receipt by Payer (With Multiple Imputations (5) for Family Income and Home Assets). (PDF 81 kb) [file 12913_2015_978_MOESM1_ESM.pdf]

Additional File 1. Odds Ratios From Logistic Regression Models Explaining Home Care Receipt by Payer (With Multiple Imputations (5) for Family Income and Home Assets)

	All Sources [A]		Public Program Only: Medicare Only [B]		Public Program Only: Medicaid [C]		Personal Only [D]		Public & Personal [E]	
	Odds Ratio	(95% CI)	Odds Ratio	(95% CI)	Odds Ratio	(95% CI)	Odds Ratio	(95% CI)	Odds Ratio	(95% CI)
Need for Caregiving										
No. of ADL's										
1-2	1.11	(0.96-1.29)	2.54 **	(1.51-4.29)	1.44	(0.91-2.26)	0.96	(0.82-1.13)	1.60	(0.99-2.57)
3-4	1.88 **	(1.60-2.21)	5.05 **	(2.99-8.54)	2.68 **	(1.68-4.25)	1.37 **	(1.14-1.64)	2.90 **	(1.78-4.71)
5-6	3.21 **	(2.65-3.89)	6.30 **	(3.62-10.96)	3.35 **	(2.01-5.58)	1.96 **	(1.57-2.45)	6.70 **	(4.05-11.09)
No. of IADL's										
2-3	2.37 **	(2.02-2.78)	2.46 **	(1.41-4.29)	2.35 **	(1.35-4.12)	2.28 **	(1.91-2.72)	2.05 **	(1.20-3.51)
4-5	4.21 **	(3.53-5.04)	4.74 **	(2.72-8.24)	5.69 **	(3.27-9.91)	3.56 **	(2.91-4.35)	3.21 **	(1.87-5.52)
6-8	5.85 **	(4.82-7.11)	5.99 **	(3.39-10.58)	9.46 **	(5.32-16.83)	4.22 **	(3.37-5.27)	4.19 **	(2.41-7.30)
Age	1.02 **	(1.01-1.03)	0.99	(0.98-1.01)	0.98 **	(0.96-0.99)	1.03 **	(1.02-1.04)	1.02 *	(1.00-1.03)
Demographics										
Female	0.95	(0.85-1.06)	0.86	(0.67-1.11)	0.93	(0.71-1.22)	0.91	(0.80-1.04)	1.22	(0.94-1.57)
Race										
African American	0.68 **	(0.56-0.82)	1.04	(0.73-1.48)	1.67 **	(1.19-2.32)	0.43 **	(0.33-0.56)	0.72	(0.49-1.06)
Other	0.82	(0.49-1.38)	1.01	(0.34-2.98)	0.88	(0.34-2.27)	0.93	(0.50-1.74)	0.28	(0.04-2.06)
Hispanic	1.00	(0.78-1.29)	0.70	(0.38-1.27)	1.30	(0.82-2.08)	1.00	(0.73-1.37)	0.73	(0.41-1.31)
Economic Resources										
Education										
High school grad	1.42 **	(1.27-1.60)	0.97	(0.75-1.27)	0.55 **	(0.41-0.76)	1.76 **	(1.55-2.01)	1.13	(0.86-1.47)
College degree	2.10 **	(1.73-2.55)	0.97	(0.57-1.64)	0.13 **	(0.04-0.42)	2.71 **	(2.22-3.32)	1.94 **	(1.27-2.98)
Family income										
15,000-29,999	1.07	(0.92-1.24)	0.76	(0.56-1.03)	0.32 **	(0.20-0.52)	1.55 **	(1.32-1.83)	0.67 *	(0.48-0.94)
30,000-49,999[a]	1.08	(0.87-1.33)	0.51	(0.25-1.03)	0.27 **	(0.11-0.66)	1.61 **	(1.29-2.02)	0.59	(0.33-1.07)
50,000-74,999	1.25	(0.91-1.70)	0.63	(0.26-1.52)	(var omitted)[a]		1.98 **	(1.37-2.87)	0.57	(0.28-1.15)
75,000+	1.82 **	(1.29-2.57)	0.44	(0.08-2.46)	(var omitted)[a]		2.89 **	(2.01-4.16)	0.40	(0.08-1.91)
Home assets										
< 150,000	0.74 **	(0.64-0.86)	1.06	(0.79-1.43)	0.48 **	(0.33-0.70)	0.84	(0.69-1.04)	0.60 **	(0.44-0.81)
≥ 150,000	0.79	(0.60-1.05)	0.87	(0.53-1.43)	0.23 **	(0.09-0.63)	0.96	(0.71-1.29)	0.74	(0.45-1.21)
Year										
1994	1.08	(0.94-1.23)	3.39 **	(2.46-4.66)	0.94	(0.67-1.32)	1.08	(0.93-1.26)	0.32 **	(0.23-0.43)
1999	0.53 **	(0.46-0.62)	1.90 **	(1.32-2.75)	1.00	(0.70-1.42)	0.45 **	(0.38-0.54)	0.38 **	(0.27-0.52)
2004	0.36 **	(0.31-0.42)	0.85	(0.55-1.31)	1.00	(0.69-1.45)	0.33 **	(0.27-0.40)	0.17 **	(0.11-0.26)
Informal Resources										
Informal Care Hours										
0	4.35 **	(3.67-5.15)	1.28	(0.92-1.79)	3.58 **	(2.45-5.22)	5.18 **	(4.20-6.38)	1.24	(0.88-1.75)
1-8	2.51 **	(2.11-2.98)	1.14	(0.81-1.62)	2.25 **	(1.51-3.37)	3.06 **	(2.47-3.79)	1.51 *	(1.07-2.13)
9-24	1.34 **	(1.11-1.60)	1.00	(0.71-1.43)	1.18	(0.74-1.87)	1.60 **	(1.27-2.02)	1.00	(0.70-1.44)
Marital Status										
Single	1.13	(0.86-1.48)	0.67	(0.36-1.24)	1.35	(0.70-2.60)	1.39 *	(1.02-1.90)	0.77	(0.42-1.38)
Widowed	1.53 **	(1.34-1.75)	0.93	(0.69-1.27)	1.37	(0.93-2.01)	1.75 **	(1.50-2.05)	0.94	(0.69-1.27)
Divorced	1.59 **	(1.28-1.97)	1.23	(0.78-1.95)	2.25 **	(1.40-3.62)	1.55 **	(1.20-2.00)	0.76	(0.45-1.30)
Number of Children										
1	0.71 **	(0.60-0.85)	0.53 **	(0.36-0.78)	0.63 *	(0.41-0.97)	0.82 *	(0.67-0.99)	0.88	(0.61-1.28)
2	0.70 **	(0.59-0.82)	0.52 **	(0.36-0.75)	0.58 *	(0.38-0.89)	0.87	(0.72-1.04)	0.59 **	(0.40-0.86)
3	0.64 **	(0.54-0.77)	0.55 **	(0.37-0.82)	0.79	(0.51-1.22)	0.70 **	(0.57-0.86)	0.72	(0.48-1.07)
4+	0.62 **	(0.53-0.74)	0.58 **	(0.41-0.82)	1.06	(0.73-1.54)	0.60 **	(0.49-0.74)	0.70	(0.48-1.02)

Notes: NLTCS, 1989-2004 (*N* = 12,956). *p<.05, **p<.01. Models include state fixed effects.

[a]Family income was topcoded at $30,000+ in the Medicaid model due to the very small number of respondents with incomes over $30,000 receiving services financed by Medicaid. Therefore, the second income dummy variable gives the difference in the outcome between those with incomes over $30,000 and those in the lowest income category.
