# Supplementary material for: Who pays for home care? A study of nationally representative data on disabled older Americans
Source: BMC Health Serv Res. 2015 Jul 31;15:301. doi: 10.1186/s12913-015-0978-x (PMC4521465; doi:10.1186/s12913-015-0978-x)
Supplement: Additional file 2: — OLS Regression Models Explaining Log Hours of Home Care by Payer (With Multiple Imputations (5) for Family Income and Home Assets). (PDF 81 kb) [file 12913_2015_978_MOESM2_ESM.pdf]

Additional File 2. OLS Regression Models Explaining Log Hours of Home Care by Payer (With Multiple Imputations (5) for Family Income and Home Assets)

	All Sources [A]		Public Program Only				Personal Only [D]		Public & Personal [E]	
			Medicare Only [B]		Medicaid [C]					
	Coef.	S.E.	Coef.	S.E.	Coef.	S.E.	Coef.	S.E.	Coef.	S.E.
Need for Caregiving										
No. of ADL's										
1-2	.02	(.09)	.43	(.38)	.61 *	(.28)	-.04	(.10)	-.72	(.41)
3-4	.37 **	(.10)	.44	(.38)	.77 **	(.29)	.24 *	(.11)	-.27	(.40)
5-6	.95 **	(.11)	.72	(.39)	.77 *	(.32)	1.11 **	(.13)	.19	(.41)
No. of IADL's										
2-3	.23 *	(.10)	-.20	(.39)	.06	(.33)	.28 *	(.12)	.09	(.50)
4-5	.56 **	(.11)	.15	(.39)	.03	(.33)	.49 **	(.13)	.46	(.48)
6-8	1.12 **	(.12)	.29	(.40)	.54	(.35)	1.22 **	(.14)	.77	(.47)
Age	.01 *	(.00)	-.01	(.01)	.01	(.01)	.01 **	(.00)	.00	(.01)
Demographics										
Female	.04	(.06)	-.02	(.15)	.11	(.16)	.04	(.07)	-.03	(.20)
Race										
African American	.06	(.10)	.69 **	(.21)	.09	(.20)	-.31	(.16)	.12	(.29)
Other	-.29	(.30)	-.59	(.67)	.12	(.58)	-.05	(.39)	-1.41	(1.54)
Hispanic	.42 **	(.13)	.95 *	(.37)	.00	(.26)	.40 *	(.18)	.51	(.48)
Economic Resources										
Education										
High school grad	.03	(.06)	.09	(.16)	.04	(.18)	.05	(.08)	-.22	(.22)
College degree	.14	(.10)	.05	(.39)	.43	(.88)	.17	(.11)	-.28	(.36)
Family income										
15,000-29,999	.01	(.08)	-.12	(.21)	.41	(.28)	.04	(.09)	.06	(.26)
30,000-49,999[a]	.08	(.12)	.02	(.45)	-.29	(.70)	.07	(.14)	.13	(.45)
50,000-74,999	.02	(.18)	.03	(.55)	(var omitted)[a]		-.09	(.17)	.62	(.59)
75,000+	.58 **	(.19)	-.30	(.89)	(var omitted)[a]		.61 **	(.19)	.19	(1.18)
Home assets										
< 150,000	-.03	(.06)	.00	(.20)	-.10	(.19)	-.03	(.09)	-.01	(.22)
≥ 150,000	.12	(.10)	.15	(.38)	-.33	(.55)	.17	(.13)	.03	(.34)
Year										
1994	.01	(.07)	.19	(.20)	-.05	(.19)	-.07	(.09)	.37	(.25)
1999	-.09	(.08)	.03	(.23)	-.30	(.20)	-.12	(.10)	.36	(.25)
2004	-.16	(.08)	.28	(.29)	-.30	(.21)	-.28 **	(.10)	.68 *	(.34)
Informal Resources										
Informal Care Hours										
9-24	.37 **	(.10)	.12	(.23)	-.03	(.28)	.36 **	(.13)	.24	(.29)
1-8	.58 **	(.09)	.37	(.24)	.13	(.23)	.39 **	(.12)	.45	(.28)
0	.76 **	(.09)	.64 **	(.22)	.65 **	(.23)	.59 **	(.12)	.65 *	(.29)
Marital Status										
Single	.07	(.14)	.01	(.38)	.49	(.38)	-.02	(.16)	-.23	(.45)
Widowed	.16 *	(.07)	-.21	(.21)	.32	(.23)	.20 *	(.09)	.06	(.24)
Divorced	.04	(.12)	.02	(.29)	.10	(.30)	-.07	(.15)	-.12	(.45)
Number of Children										
1	-.10	(.09)	.55 *	(.24)	-.01	(.27)	-.18	(.11)	.07	(.30)
2	-.04	(.08)	.22	(.22)	.64 **	(.24)	-.13	(.10)	-.24	(.30)
3	.00	(.09)	.44	(.25)	.06	(.24)	-.07	(.12)	.29	(.33)
4+	-.06	(.09)	.48 *	(.23)	.10	(.21)	-.19	(.11)	.16	(.30)

Notes: NLTCS, 1989-2004 (all sources model *N* = 2,428, Medicare only *N* = 335, Medicaid *N* = 313, personal only *N* = 1,621, public & personal *N* = 326). *p<.05, **p<.01. Models include state fixed effects.

[a]Family income was topcoded at $30,000+ in the Medicaid model due to the very small number of respondents with incomes over $30,000 receiving services financed by Medicaid. Therefore, the second income dummy variable gives the difference in the outcome between those with incomes over $30,000 and those in the lowest income category.
