# Supplementary material for: Who pays for home care? A study of nationally representative data on disabled older Americans
Source: BMC Health Serv Res. 2015 Jul 31;15:301. doi: 10.1186/s12913-015-0978-x (PMC4521465; doi:10.1186/s12913-015-0978-x)
Supplement: Additional file 3: — Descriptive Statistics for Home Care Services Use by Payer. (PDF 47 kb) [file 12913_2015_978_MOESM3_ESM.pdf]

Additional File 3. Descriptive Statistics for Home Care Services Use by Payer

	Receiving at Least One Hour		Conditional on Receiving at Least One Hour				
Payer	Number	Percent	Mean	Median	Min.	Max.	Skewness
Any Payer	2428	20.7	20	6	1	336	4.0
Medicare Only	335	2.9	11	4	1	108	3.4
Medicaid	313	2.7	19	12	1	168	2.7
Personal Only	1,621	13.8	20	5	1	336	4.0
Public & Personal	326	2.8	19	5	1	288	3.6

Notes: NLTCS, 1989-2004 (*N* = 11,725).
