# Supplementary figures and images for: Who pays for home care? A study of nationally representative data on disabled older Americans
Source: BMC Health Serv Res. 2015 Jul 31;15:301. doi: 10.1186/s12913-015-0978-x (PMC4521465; doi:10.1186/s12913-015-0978-x)

Additional File 4. Care Hours Received From Each Payer by Family Income

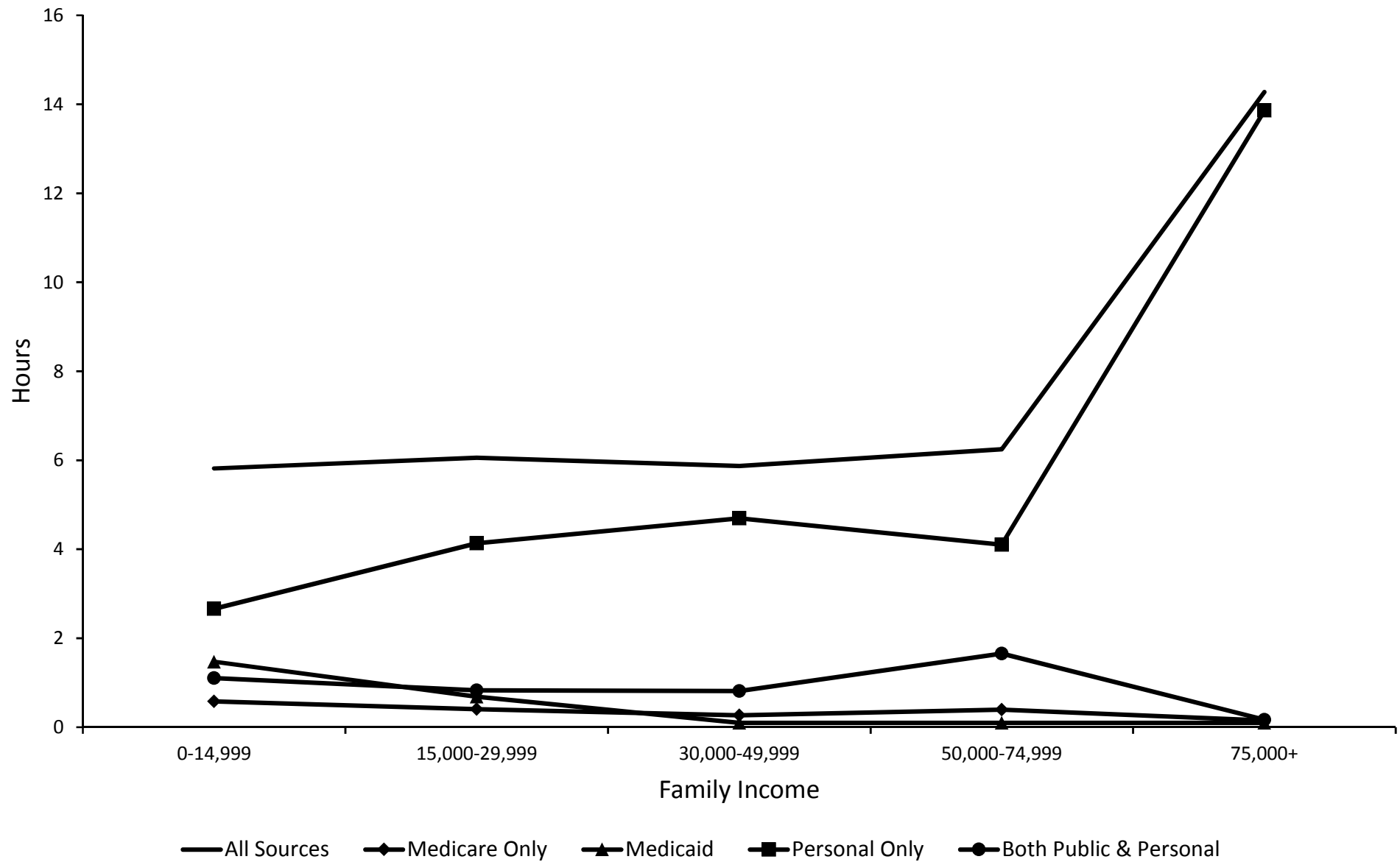

Supplement: Additional file 4: — Care Hours Received From Each Payer by Family Income. (PDF 24 kb) [file 12913_2015_978_MOESM4_ESM.pdf]

Additional File 5. Care Hours Received From Each Payer by Number of ADL's

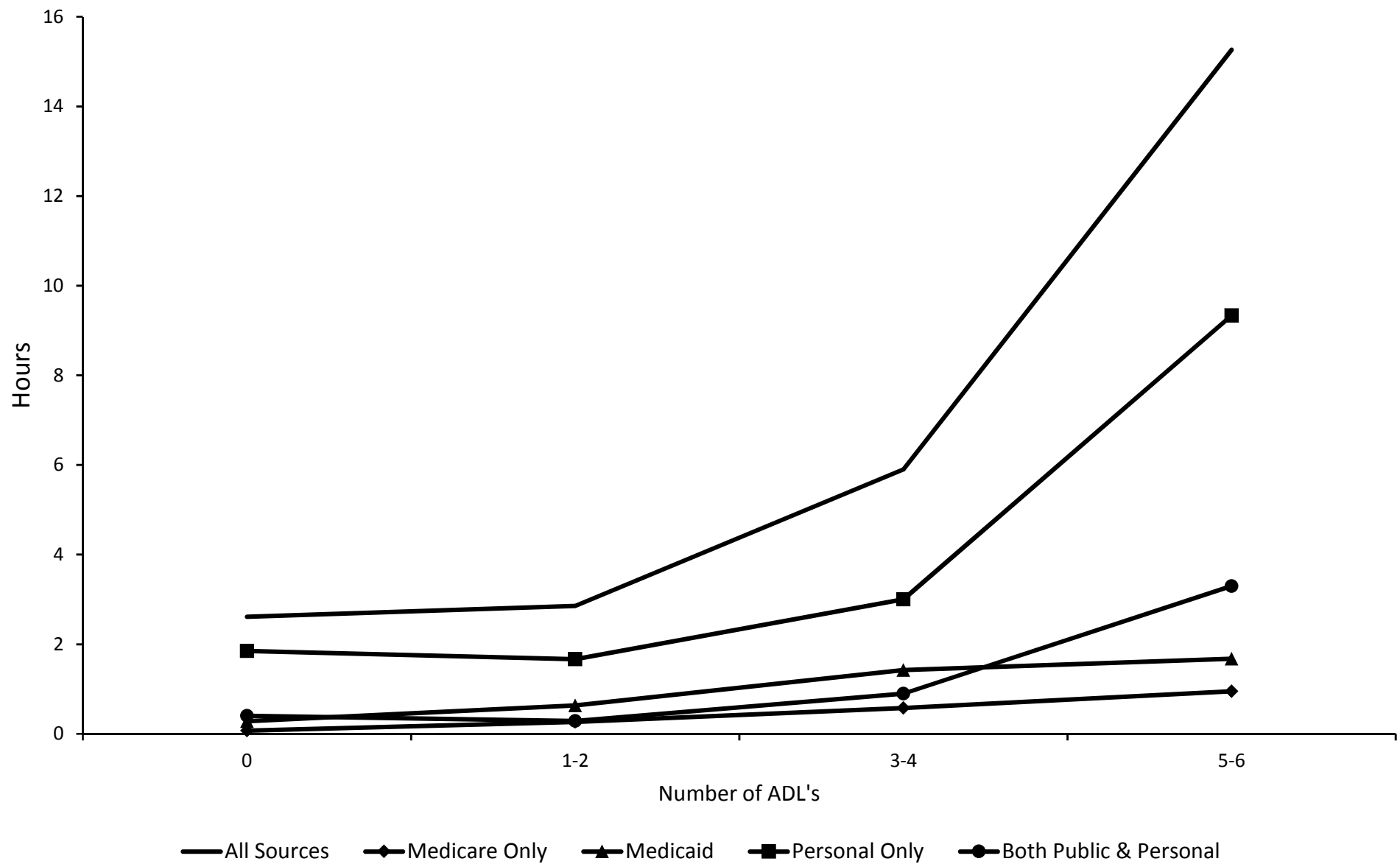

Supplement: Additional file 5: — Care Hours Received From Each Payer by Number of ADL’s. (PDF 24 kb) [file 12913_2015_978_MOESM5_ESM.pdf]
