# Supplementary material for: Who pays for home care? A study of nationally representative data on disabled older Americans
Source: BMC Health Serv Res. 2015 Jul 31;15:301. doi: 10.1186/s12913-015-0978-x (PMC4521465; doi:10.1186/s12913-015-0978-x)
Supplement: Additional file 6: — Odds Ratios From Logistic Regression Models Explaining Receipt of Care Provided by Caregivers Paid by Medicaid Alone. (PDF 76 kb) [file 12913_2015_978_MOESM6_ESM.pdf]

Additional File 6. Odds Ratios From Logistic Regression Models Explaining Home Care Receipt Paid for by Medicaid Alone

	Odds Ratio		(95% CI)
Need for Caregiving			
No. of ADL's			
1-2	1.34		(0.74 - 2.42)
3-4	2.26	**	(1.27 - 4.01)
5-6	2.39	**	(1.25 - 4.57)
No. of IADL's			
2-3	3.12	**	(1.34 - 7.26)
4-5	6.38	**	(2.73 - 14.92)
6-8	12.00	**	(5.18 - 27.81)
Age	0.99		(0.96 - 1.01)
Basic Demographic Characteristics			
Female	0.93		(0.65 - 1.34)
Race			
African American	1.71	*	(1.11 - 2.63)
Other	1.06		(0.33 - 3.44)
Hispanic	0.82		(0.41 - 1.66)
Economic Resources			
Education			
High school grad	0.60	**	(0.41 - 0.88)
College degree	0.20	*	(0.06 - 0.68)
Family income			
15,000-29,999	0.23	**	(0.13 - 0.41)
30,000+	0.19	**	(0.06 - 0.64)
Family income missing	0.32	**	(0.20 - 0.52)
Home assets			
Yes, home value missing	0.56	**	(0.37 - 0.86)
Home value < 150,000	0.38	**	(0.24 - 0.59)
Home value ≥ 150,000	0.19	**	(0.06 - 0.62)
Year			
1994	1.07		(0.68 - 1.68)
1999	1.06		(0.65 - 1.71)
2004	0.91		(0.57 - 1.47)
Informal Caregiving Resources			
Hours of Informal Care			
0	4.27	**	(2.55 - 7.15)
1-8	3.14	**	(1.81 - 5.46)
9-24	1.31		(0.69 - 2.48)
Marital Status			
Single	1.10		(0.49 - 2.49)
Widowed	1.00		(0.62 - 1.61)
Divorced	1.85	*	(1.07 - 3.21)
Number of Children			
1	0.62		(0.35 - 1.09)
2	0.60		(0.35 - 1.01)
3	0.75		(0.42 - 1.34)
4+	1.04		(0.64 - 1.69)

Notes: NLTCS, 1989-2004 (N = 11.725). Model includes state fixed effects. *p <.05, **p <.01.
