# Supplementary material for: Who pays for home care? A study of nationally representative data on disabled older Americans
Source: BMC Health Serv Res. 2015 Jul 31;15:301. doi: 10.1186/s12913-015-0978-x (PMC4521465; doi:10.1186/s12913-015-0978-x)
Supplement: Additional file 7: — Odds Ratios From Logistic Regression Models Explaining Receipt of Care Provided by Caregivers Paid by Family Members. (PDF 76 kb) [file 12913_2015_978_MOESM7_ESM.pdf]

Additional File 7. Odds Ratios From Logistic Regression Models Explaining Home Care Receipt Paid for by Family Members

	Odds Ratio		(95% CI)
Need for Caregiving			
No. of ADL's			
1-2	2.56	**	(1.33 - 4.90)
3-4	2.85	**	(1.39 - 5.86)
5-6	5.09	**	(2.44 - 10.61)
No. of IADL's			
2-3	1.26		(0.70 - 2.27)
4-5	1.93	*	(1.03 - 3.64)
6-8	3.76	**	(1.98 - 7.14)
Age	1.04	**	(1.02 - 1.06)
Basic Demographic Characteristics			
Female	0.85		(0.60 - 1.22)
Race			
African American	0.47	*	(0.24 - 0.94)
Other	0.90		(0.18 - 4.40)
Hispanic	0.82		(0.39 - 1.75)
Economic Resources			
Education			
High school grad	1.38		(0.96 - 1.99)
College degree	2.20	**	(1.32 - 3.66)
Family income			
15,000-29,999	0.96		(0.59 - 1.55)
30,000-49,999	0.89		(0.42 - 1.88)
50,000-74,999	1.82		(0.88 - 3.74)
75,000+	2.64	*	(1.16 - 6.04)
Family income missing	1.69	**	(1.17 - 2.44)
Home assets			
Yes, home value missing	0.67		(0.43 - 1.05)
Home value < 150,000	0.56	**	(0.37 - 0.83)
Home value ≥ 150,000	0.65		(0.40 - 1.05)
Year			
1994	0.42	**	(0.27 - 0.65)
1999	0.34	**	(0.21 - 0.55)
2004	0.41	**	(0.27 - 0.63)
Informal Caregiving Resources			
Hours of Informal Care			
0	1.40		(0.88 - 2.23)
1-8	1.53		(0.96 - 2.44)
9-24	0.86		(0.50 - 1.47)
Marital Status			
Single	0.90		(0.36 - 2.21)
Widowed	1.03		(0.68 - 1.58)
Divorced	1.19		(0.61 - 2.33)
Number of Children			
1	0.82		(0.46 - 1.46)
2	1.49		(0.90 - 2.46)
3	1.29		(0.74 - 2.22)
4+	1.16		(0.68 - 1.98)

Notes: NLTCS, 1989-2004 (N = 11.725). Model includes state fixed effects. *p <.05, **p <.01.
